# The adipokine vaspin is associated with decreased coronary in-stent restenosis *in vivo* and inhibits migration of human coronary smooth muscle cells *in vitro*

**DOI:** 10.1371/journal.pone.0232483

**Published:** 2020-05-11

**Authors:** Stefan P. Kastl, Katharina M. Katsaros, Konstantin A. Krychtiuk, Gerlinde Jägersberger, Christoph Kaun, Kurt Huber, Johann Wojta, Walter S. Speidl

**Affiliations:** 1 Department of Internal Medicine II, Medical University of Vienna, Vienna, Austria; 2 The Ludwig Boltzmann Cluster for Cardiovascular Research, Vienna, Austria; 3 Department of Medicine (Cardiology and Emergency Medicine), Wilhelminenhospital, Vienna, Austria; INSERM, Université de Bordeaux, FRANCE

## Abstract

**Background:**

Percutaneous coronary intervention represents the most important treatment modality of coronary artery stenosis. In-stent restenosis (ISR) is still a limitation for the long-term outcome despite the introduction of drug eluting stents. It has been shown that adipokines directly influence vessel wall homeostasis by influencing the function of endothelial cells and arterial smooth muscle cells. Visceral adipose tissue-derived serpin vaspin was recently identified as a member of serine protease inhibitor family and serveral studies could demonstrate a relation to metabolic diseases. The aim of this study was to investigate a role of vaspin in the development of in-stent restenosis *in vivo* and on migration of smooth muscle cells and endothelial cells *in vitro*.

**Methods:**

We studied 85 patients with stable coronary artery disease who underwent elective and successful PCI with implatation of drug eluting stents. Blood samples were taken directly before PCI. Vaspin plasma levels were measured by specific ELISA. ISR was evaluated eight months later by coronary angiography. Human coronary artery smooth muscle cells (HCASMC) and human umbilical vein endothelial cells (HUVEC) migration was analyzed by an *in-vitro* migration assay with different concentrations (0.004ng/mL up to 40ng/mL) of vaspin as well as by an scratch assay. For proliferation an impedance measurement with specialiced E-Plates was performed.

**Results:**

During the follow up period, 14 patients developed ISR. Patients with ISR had significantly lower vaspin plasma levels compared to patients without ISR (0.213 ng/ml vs 0.382 ng/ml; p = 0.001). In patients with plasma vaspin levels above 1.35 ng/ml we could not observe any restenosis. There was also a significant correlation of plasma vaspin levels and late lumen loss in the stented coronary segments. Further we could demonstrate that vaspin nearly abolishes serum induced migration of HCASMC (100% vs. 9%; p<0.001) in a biphasic manner but not migration of HUVEC. Proliferation of HCASMC and HUVEC was not modulated by vaspin treatment.

**Conclusion:**

We were able to show that the adipokine vaspin selectively inhibits human coronary SMC migration *in vitro* and has no effect on HUVEC migration. Vaspin had no effect on proliferation of HUVEC which is an important process of the healing of the stented vessel. In addition, the occurrence of ISR after PCI with implantation of drug eluting stents was significantly associated with low vaspin plasma levels before intervention. Determination of vaspin plasma levels before PCI might be helpful in the identification of patients with high risk for development of ISR after stent implantation. In addition, the selective effects of vaspin on smooth muscle cell migration could potentially be used to reduce ISR without inhibition of re-endothelialization of the stented segment.

## Introduction

Percutaneous coronary intervention (PCI) represents the most important treatment modality of coronary artery stenosis. However the occurrence of In-stent restenosis (ISR) is still a limitation for the long-term outcome despite the introduction of drug eluting stents (DES).[1] Several contributory factors have been identified during the last years, but the overall underlying mechanisms are still unclear.

ISR occurs at different points in time (early and late restenosis) after implantation of a DES and involves numerous cellular and molecular mechanisms. [2,3] The pathophysiology of restenosis involves accumulation of new tissue within the arterial wall. Smooth muscle cell (SMC) migration and extracellular matrix secretion (ECM) plays a central role in neointimal hyperplasia (NIH), which is nowadays seen as pathognomic of ISR.[4] ECM synthesis by these SMCs is responsible for the increasing volume of intimal tissue, which is composed of ECM proteoglycans and collagens.[5] Over the months after the implantation of a DES there is a shift towards greater ECM synthesis rather than SMC proliferative activity.[6,7] So inhibition of initial SMC migration to the media could play a key role in later NIH. But unlike the proliferative aspect of the SMCs, little is known about their ‘‘motile” activity after stent-implantation, which allows them to migrate into the media. The use of DES reduced the incidence of restenosis but the cytostatic agents also delayed endothelialization of the implanted stent which plays a key role for the long-term outcome. Incomplete endothelialization can lead to stent-thrombosis and acute myocardial infarction after stopping of antiplatelet therapy.

Vaspin is an adipocytokine that has been isolated from the visceral adipose tissue of Otsuka Long-Evans Tokushima Fatty (OLETF) rats, which is a diabetes rat model.[8] Due to the fact that vaspin is characterized by the presence of a core domain consisting of three β-sheets and nine α-helices it is likely that vaspin belongs to the serine protease inhibitors (serpin) family. [9,10] But still nothing is known about the physiologic inhibitory function of vaspin. Several Studies in OLETF could demonstrate that vaspin production decreased as diabetes worsened but increased by treatment with insulin or pioglitazone.[11] This suggests that the up-regulation of vaspin may have a defensive action against insulin resistance. A recent study could clearly show that vaspin is also produced by periadventitial adipose tissue which may play an important paracrine role during the development of ISR.[12]

Therefore we tested whether plasma levels of vaspin are related to the clinical manifestation of ISR in patient with stable coronary artery disease that have received an DES and if vaspin inhibits the migration of smooth muscle cells and endothelial cells *in vitro*. Further, we wanted to know if vaspin has any negative effects on the healing process (endothelialization) of the stented vessel.

## Methods

### Patients

Blood samples were taken prospectively from all patients with stable coronary artery disease who were scheduled for elective PCI. The type, number, length, and size of the stent(s) implanted were left to the discretion of the interventionalist. All patients with DES only (n = 107) were asked to participate in this study, and we included all 85 patients who gave their informed consent for follow-up angiography.

Paclitaxel-eluting stents (Taxus; Boston Scientific, Boston, MA, USA) were implanted in 62 patients (72.9%); sirolimus-eluting stents (Cypher; Cordis, Johnson & Johnson, Miami Lakes, FL, USA) were used in 23 patients (27.1%). The study was approved by the institutional ethics committee (Ethikkommission der Medizinischen Universität Wien). Patients with concurrent severe illness, acute coronary syndrome within three months before angioplasty, PCI for restenosis and unsuccessful procedure were excluded. Clopidogrel therapy was either started on the day before angiography (n = 56) or immediately after stent implantation (n = 29) with four tablets (300 mg). During the intervention, all patients were treated with unfractionated heparin. After the procedure, patients were maintained on 100 mg aspirin indefinitely, 75 mg clopidogrel for at least 6 months. Other medications were given in accordance with the relevant guidelines and regulations.

### Angiographic definitions

Quantitative coronary angiographic analysis was performed by a single, experienced researcher who was blinded to clinical characteristics and laboratory measurements. The modified American College of Cardiology/American Heart Association grading system (types A, B1, B2, and C) was used to characterize lesion morphology. The off-line quantitative coronary angiographic analysis was performed with an automated edge-detection system (QCA-CMS V 6.0; Medis, Medical Imaging Systems). A contrast-filled nontapered catheter tip was used for calibration. The reference diameter was measured by interpolation. The angiographic parameters that have been measured were vessel size maximal balloon pressure (atm), balloon-to-vessel ratio, lesion length, and length of stented segment. Minimal luminal diameter (prior and post procedure) and diameter stenosis (prior and post procedure) have been measured in-stent and in-segment (including the stented segment, as well as both 5mm margins proximal and distal to the stent). Angiographic restenosis was evaluated at six to eight months follow-up, or earlier if clinically indicated. The primary end point of the study was angiographic restenosis (diameter stenosis of at least 50% based on in-segment analysis) at follow-up angiography. The secondary end points were in-stent LL and the need for target lesion revascularization due to restenosis in the presence of symptoms or objective signs of ischemia during follow-up.

### Blood sampling

After informed consent was obtained, blood samples were taken under fasting conditions. Samples were taken in the morning before PCI (before treatment with unfractionated heparin). Therefore, venous blood was drawn from the antecubital vein with minimal tourniquet pressure into EDTA tubes. After centrifugation (4°C; 660g for 25 min) plasma samples were stored at -80°C until use.

### Cell culture experiments

Human coronary artery smooth muscle cells (HCASMC) were isolated from pieces of coronary arteries obtained from patients undergoing heart transplantation. Such smooth muscle cells were cultured and characterized as already described.[13] Human umbilical vein endothelial cells (HUVEC) were isolated from fresh umbilical cords by mild collagenase treatment, and cultivated as described.[14]

### Cell migration assay

The migration of HCASMC and HUVEC was examined using a colorimetric cell migration assay (Millipore, Billerica, USA) based on the Boyden chamber principle using inserts with a pore size of 8 μm.

HCASMC and HUVEC were trypsinized, washed 2x with PBS, resuspended in 1% FBS in M199 in the presence or absence of vaspin of different concentrations (0.004ng/mL up to 4ng/mL), and afterwards added to the upper tray (2.5x10^4^cells/300μL). M199 with 10% FBS and the same concentration of vaspin as in the upper tray was added to the bottom chamber. As a negative control we used serum free M199 in both chambers. After 6 hours at 37°C, nonmigrating cells were scraped from the upper surface of the filter. Cells on the bottom surface were incubated with Cell Stain Solution (Millipore, Billerica, USA), then subsequently extracted and detected by spectrophotometry (absorbance at 560 nm).

### Scratch assay

HCASMC and HUVEC were trypsinized, washed 2x with PBS and resuspended in 1% FBS in M199. Cells were seeded into 6-well plates at a concentratrion of 5*10^5 cells / well. After 24h the cell monolayer was "scratched" in a straight line with a 200μL pipet tip. The debris of the scratch was removed by washing the cells once with growth medium and then cells were ether stimulated with 4ng/ml vaspin or PBS as a control. After 24h remigration into the the scratched area was analyzed under the microscope. Each experiment was repeated 3 times and a representative sample is shown.

### Measurement of proliferation

Cells (HUVECs and HCASMCs) were seeded at a concentration of 2000 cells/well in the wells of an electronic microtiter plate (E-Plate®) of the xCELLigence System (ACEA Biosciences Inc., San Diego, USA). After 4h of adhesion non-adherent cells were washed away with PBS and vaspin was added at the indicated concentrations. Adhesion of cells to the gold microelectrodes impedes the flow of electric current between electrodes. This impedance value increases as cells proliferate. After 24h differences in impedance were measured.

### Determination of vaspin plasma levels

Vaspin antigen was determined by a specific enzyme-linked immunosorbent assays (ELISA), (AdipoGene, Incheon, Korea). (Sensitivity 12 pg/ml)

### Statistical analysis

Sample size calculation was based on the hypothesis that vaspin plasma levels show a difference of at least 50% in patients with and without ISR. Calculation of sample size revealed that, with an expected “real world” restenosis rate of 10%, 80 patients were needed to detect a 50% difference in vaspin levels between patients with and without ISR with a power of 80% and significance level (2-tailed) of 0.05 (15). In patients with multiple lesion interventions, only the lesion with the highest late lumen loss was included. Continuous variables are expressed as mean ± SD. Demographic data of patients with and without restenosis were compared by the unpaired Student t test. Categorical variables are summarized as counts and percentages and were compared by the chi-square or by Fisher exact test. Plasma levels of vaspin were compared by Mann-Whitney U test. Spearman's correlation was used to correlate vaspin levels with late lumen loss. Multivariate analysis was performed with the logistic regression model in which restenosis was used as dependent variable and vaspin levels as well as potentially confounding baseline variables were used as independent variables. Baseline variables were selected for the model if they appeared to be imbalanced between patients with and without restenosis indicated by a p-value <0.20. HCASMC and HUVEC migration and proliferation was analyzed by ANOVA. A value of p < 0.05 (2-tailed) was considered statistically significant. All statistical analyses were performed with the statistical software package SPSS version 11.0 (SPSS, Inc., Chicago, Illinois). The authors had full access to the data and take responsibility for its integrity. All authors have read and agree to the manuscript as written.

## Results

### Association of vaspin plasma levels with instent-restenosis

**Fig 1:**
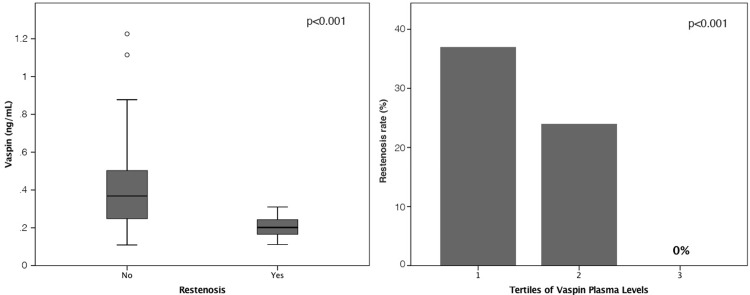
Plasma levels of vaspin before percutaneous coronary intervention (PCI) with implantation of drug-eluting stents. (A) Box plots indicate median, interquartile range (range from the 25th to the 75th percentile), and total range. p<0.001 no restenosis vs. restenosis. Restenosis rates according tertiles of vaspin plasma levels before PCI, p<0.001 (B).

**Fig 2:**
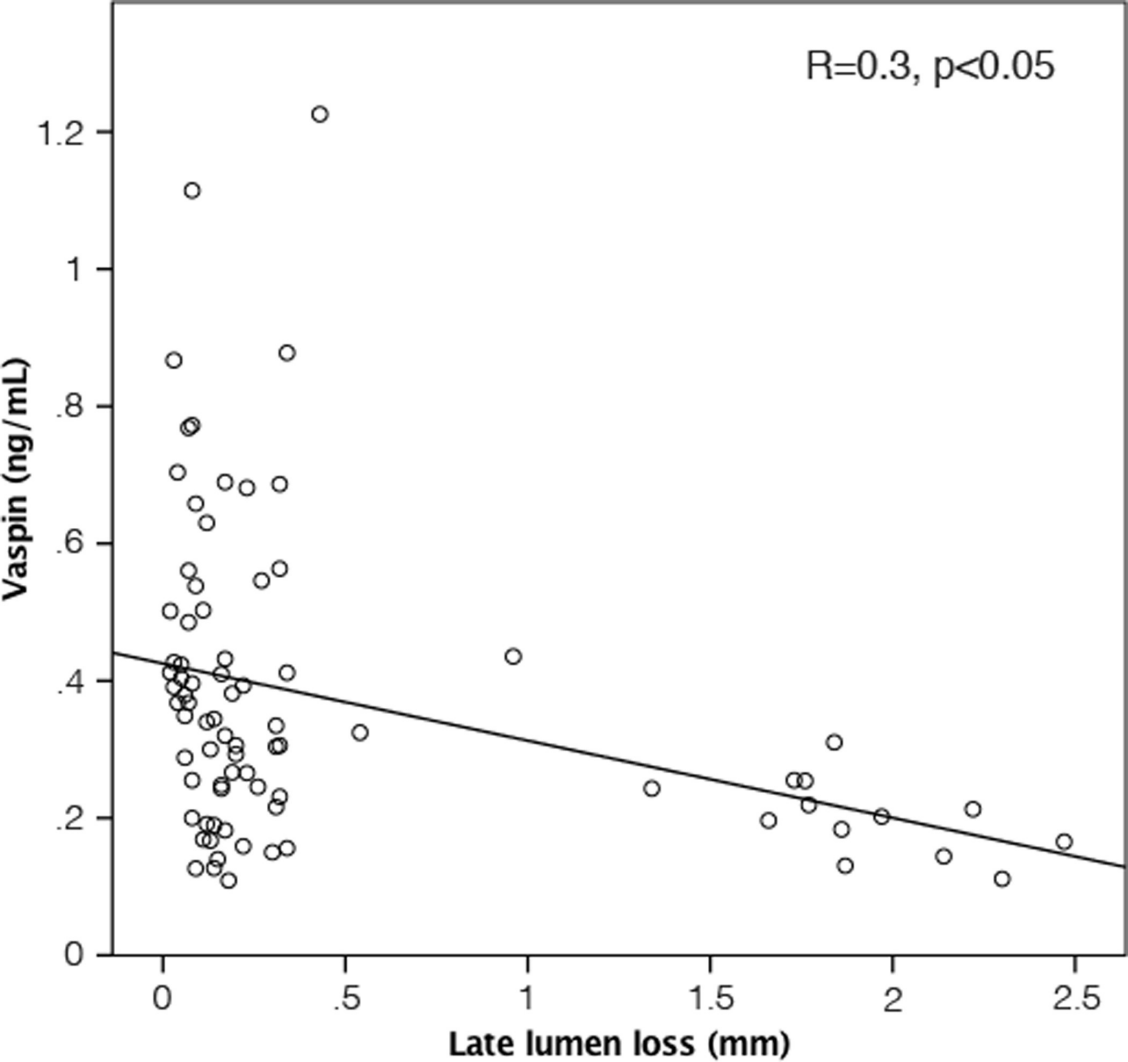
Correlation of late lumen loss with vaspin plasma levels before PCI; R = 0.3, p<0.05.

Baseline characteristics of study population are given in Table 1. The mean age was 64±10 years and 77.6% of patients were male. In-stent restenosis occurred in 14 (16.5%) patients. Angiographical and interventional characteristics of patients are given in Table 2. Patients with ISR at follow-up angiography had significantly lower vaspin plasma levels before stent-implantation compared to patients without ISR (0.213 ng/ml vs 0.382 ng/ml; p = 0.001; Fig 1A). By dividing plasma vaspin levels into tertiles we could show that the restenosis rate is also strongly related to levels of vaspin (Fig 1B). In patients with plasma vaspin levels in the third tertile before intervention we could not observe any restenosis (0%; p<0.05). Multivariate regression analysis (Table 3) revealed that a decrease of vaspin plasma levels was associated with ISR independent from clinical variables (hypertension, BMI) and procedural variables (number, diameter and type of stents). By investigating late lumen loss in the stented coronary segment we found a significant correlation with the plasma vaspin levels before intervention (R = 0.3, p<0.05; Fig 2).

**Table 1:** Baseline characteristics of patients with and without restenosis.

	Total	Restenosis	No restenosis	
	n = 85	n = 14	N = 71	p-Value
Age (years)	64±10	66±7	63±10	0.32
Sex (male)	66 (79)	9 (75)	57 (80)	0.7
Hypertension	63 (76)	7 (58)	56 (78)	0.12
Diabetes	25 (30)	3 (25)	22 (31)	0.48
Family History of CHD	46 (55)	7 (58)	39 (54)	0.54
Smoker	28 (34)	4 (33)	24 (34)	1.0
BMI (kg/m^2^)	27.9±3.7	26.6±3.6	28.1±3.7	0.19
Triglycerides (mg/dl)	175±99	159±70	177±103	0.55
TC (mg/dl)	189±43	186±27	190±45	0.81
HbA1c	6.02±0.74	6.07±0.63	6.06±0.77	0.83
Leukocytes	6.69±1.51	6.49±1.83	6.73±1.46	0.6
ACE-Inhibitors	35 (42)	7 (58)	28 (39)	0.34
ARB	10 (12)	1 (8)	9 (13)	1.0
Beta Blocker	44 (53)	8 (67)	36 (51)	0.36
Statin	64 (77)	9 (75)	55 (77)	1.0

Values are given as mean ± SD or n (%).

ACE = angiotensin-converting enzyme; ARB = angiotensin receptor blocker; BMI = body mass index; CRP = C-reactive protein; HbA1c = glycosylated hemoglobin; TC = total cholesterol.

**Table 2:** Angiographic and procedural characteristics of patients with and without restenosis.

Angiography Target vessel	Restenosis	No restenosis	
	n = 14	n = 71	p-Value
LAD	8 (57)	40 (56.3)	0.69
LCx	1 (7)	13 (18.3)	
RCA	5 (36)	18 (25.4)	
Lesion Type (A/B/C)	2/10/2	17/45/7	0.42
Vessel Size	3.23±0.39	3.26±0.37	0.79
MLD (mm)	0.68±0.21	0.67±0.23	0.99
DS (%)	78.70±7.05	80.03±7.14	0.55
Number of stents per procedure	2.33±1.37	1.78±0.92	0.08
Number of stents per vessel	1.58±0.79	1.30±0.57	0.13
**Type of Stent**			
Taxus	13 (93%)	48 (69)	0.16
Cypher	1 (7%)	22 (31)	
**Length of stented segment (mm)**	22.41±0.41	20.79±6.40	0.4
MLD after procedure			
In-stent (mm)	2.64±0.41	2.68±0.36	0.74
In-segment (mm)	2.63±0.41	2.66±0.35	0.78

LAD = left anterior descending coronary artery; LCx = left circumflex coronary artery; MLD = minimal lumen diameter; RCA = right coronary artery. MLD = minimal lumen diameter.

**Table 3:** Logistic regression model assessing the risk for in-stent restenosis after implantation of drug-eluting stents according a decrease of plasma levels of vaspin.

	Hazard ratio for 1 SD decrease of vaspin plasma levels	95% Confidence interval	p-value
Unadjusted	4.4	1.6–11.4	0.003
Adjusted for hypertension, BMI	4.2	1.5–11.6	0.005
Adjusted for stent diameter, type of stents, number of stents	6.6	1.9–23.0	0.003

BMI body mass index; SD standard deviation.

### Effect of vaspin on migration of human coronary smooth muscle cells (HCASMC)

**Fig 3:**
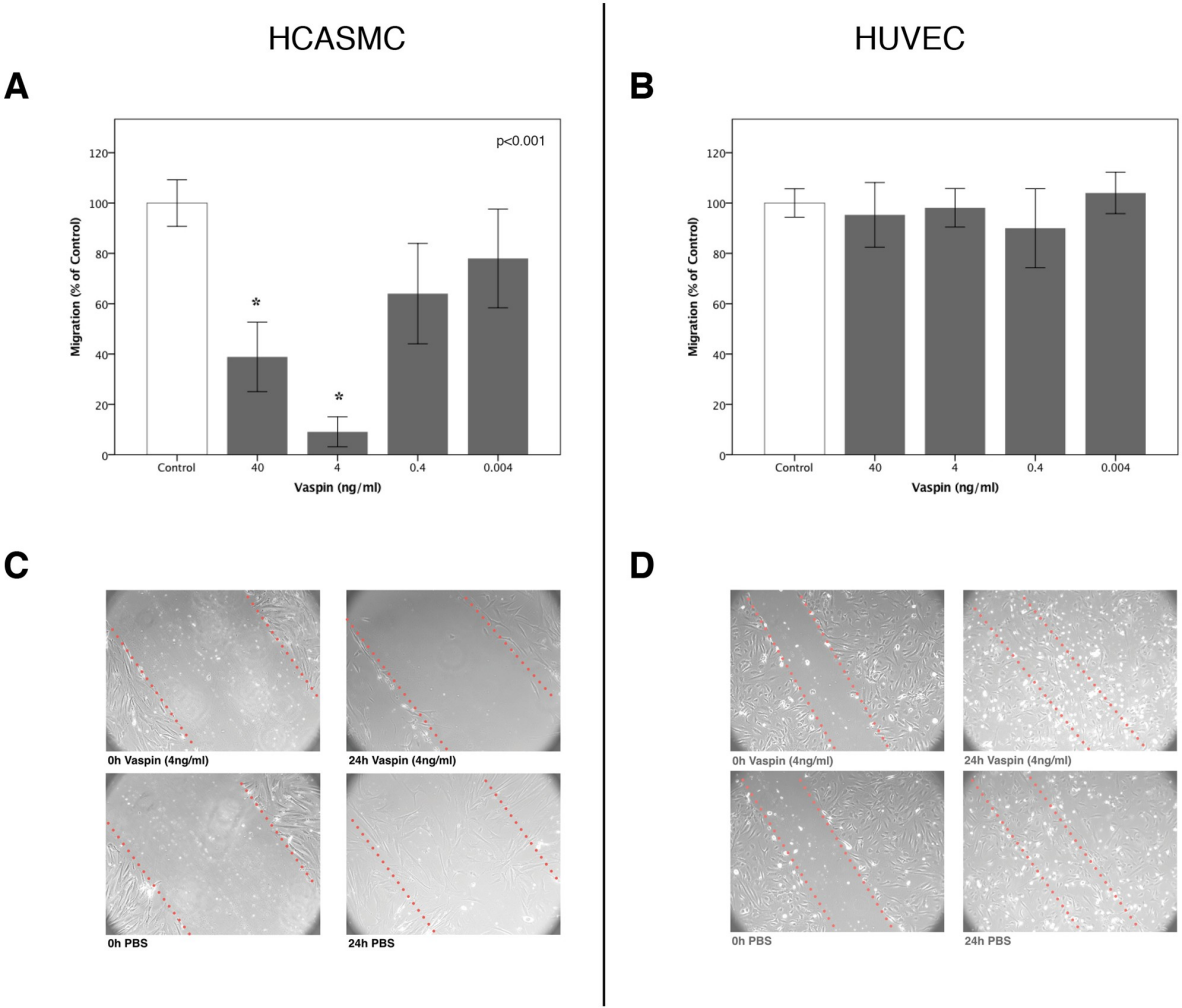
Panel A & B: Effect of vaspin on migration of human coronary smooth muscle (A), and human umbilical vein endothelial cells *in vitro* (B); * p<0.001. Panel C & D: Scratch assay to determine the effect of vaspin on wound healing migration of human coronary smooth muscle cells (C) and human umbilical vein endothelial cells (D) *in vitro*. Cells were added at a concentration of 5*10^5 cells / well. After 24h the cell monolayer was scratched in a straight line with a 200μL pipet tip. The debris of the scratch was removed by washing the cells once with growth medium and then cells were either treated with 4ng/ml vaspin (Panel A + B) or PBS as a control (Panel C + D). After 24h remigration into the scratched area was analysed under the Microscope. Each experiment was repeated 3 times and a representative sample is shown.

Treatment of HCASMC with vaspin decreased migration towards serum in a dose-dependent manner with a maximum inhibitory effect at 4 ng/mL (100±9.26% vs. 9±5.96%; p<0.001). Interestingly, the inhibitory effect of vaspin was decreased at the higher dose of 40 ng/mL resulting in a U-shaped curve (Fig 3A). In addition, vaspin (4 ng/mL) completely abolished repopulation with HCASMC in a scratch assay (Fig 3C).

### Effect of vaspin on migration of human umbilical vein endothelial cells (HUVEC)

Treatment of HUVEC with the same concentrations of vaspin was not effective in decreasing migration towards serum. It was not possible to demonstrate any effect of inhibition of migration of HUVEC at all. (100±5.65% vs. 98±7.67%; p>0.05) (Fig 3B). Also, vaspin (4 ng/mL) did not abolish repopulation with HUVEC in a scratch assay (Fig 3D).

### Effect of vaspin on the prolifferation of HUVEC and HCASMC

**Fig 4:**
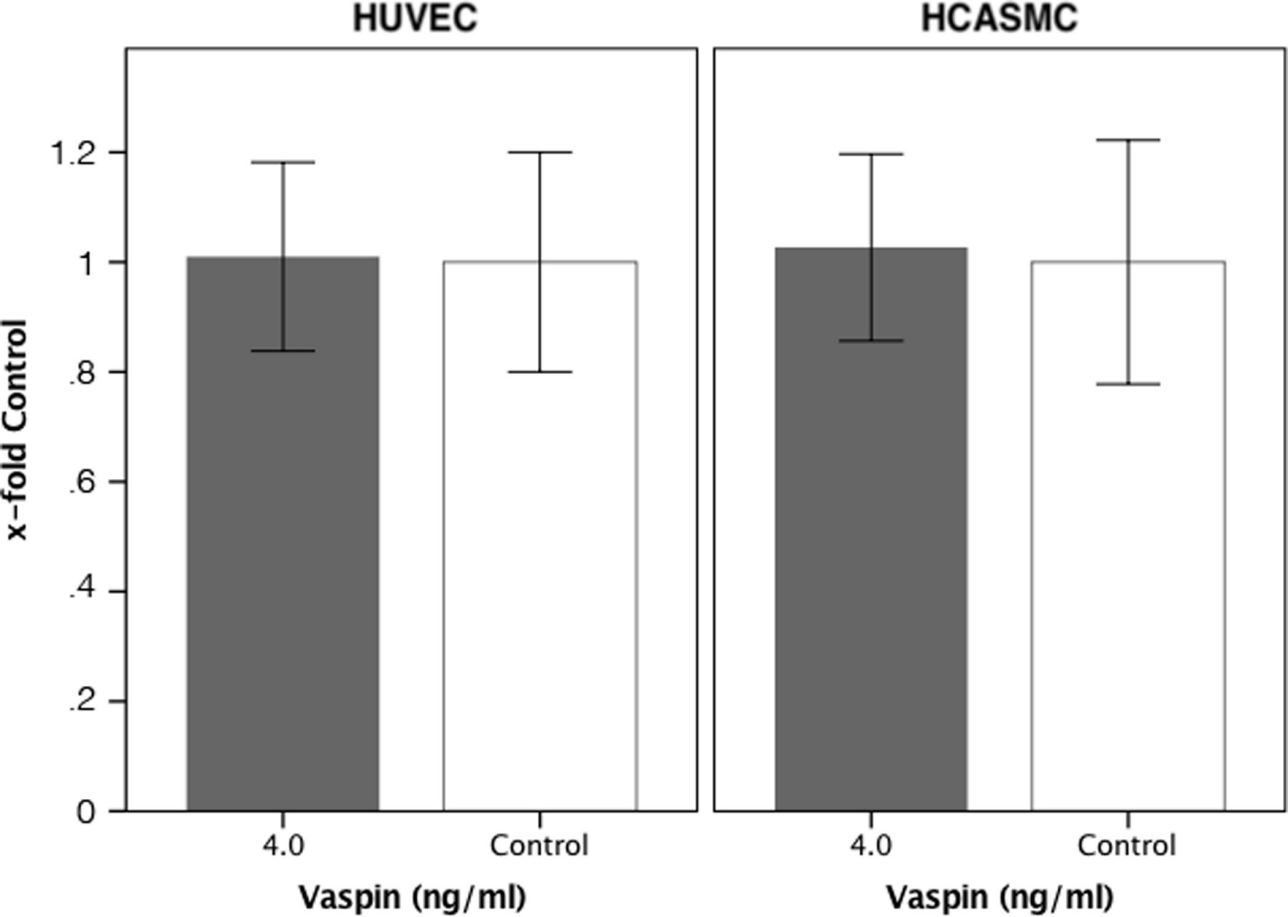
Effect of vaspin on proliferation *in vitro*. Human coronary smooth muscle cells and human umbilical vein endothelial cells were seeded at a concentration of 2000 cells/well in the wells of an electronic microtiter plate. After 24h differences in impedance, which represents proliferation, were measured.

Treatment of HUVEC and HCASMC with vaspin at a concentration of 4ng/ml for 24h had no significant effect on the proliferation of these cells compared to cells which were treated with 0ng/ml (HUVEC: 100±11% vs 103±9% p>0.05; HCASMC: 100±10% vs 101±8,6% p>0.05). (Fig 4)

## Discussion

Locally produced adipokines, especially by periadventitial adipose tissue, may affect vascular physiology and pathology.[12] It has already been described a correlation of vaspin plasma levels with carotid intima-media thickness independent of insulin resistence.[15] This indicates that vaspin may play a role in the development of atherosclerosis also in slim or non-diabetic patients. However, still little is known for the association of vaspin with atherosclerosis.[16,17] Due to the fact that intima hyperplasia induced by smooth muscle cell migration plays a key role in the development of atherosclerosis[18] as well as in the development of in-stent restenosis[19] the effects of vaspin on smooth muscle cell migration and the occurrence of ISR in correlation to the baseline plasma levels of patients with stable coronary artery disease were of special interest in our study.

We were able to show for the first time that plasma levels of vaspin are able to predict the occurrence of ISR in patients with stable CAD, independently from established CV risk factors. In addition, we could show a correlation to the late lumen loss of the stented segment of the coronary artery with plasma vaspin levels before PCI. These findings are in line with our *in-vitro* data showing a dose dependent effect on smooth muscle cell migration.

Vaspin is thought to belong to the serpin family [9,10] but the molecular target and its mode of action is not fully understood. Heiker at al. were able to determine kallikrein 7 as a first target of vaspin which could be the physiological mechanism for its compensatory actions on obesity-induced insulin resistance [20]. The effects of vaspin on smooth muscle cell migration has already been studied in an rat animal model.[21] However, due to the fact of the well described differences in gene expression patterns between different types of smooth muscle cells within one organism[22] we reinvestigated the described effect on smooth muscle cell migration in cells derived from human coronary arteries. We were able to reproduce the described inhibitory effect of vaspin on serum induced smooth muscle cell migration in a human *in vitro* model for the first time. A recent study in rat smooth muscle cells has shown that vaspin attenuates high glucose-induced proliferation and chemokinesis. In addition, it has been demonstrated that this effect was mediated by PI3K/Akt pathway as vaspin significantly attenuated Akt phosphorylation *in vitro* [23]. This in line with previous studies that demonstrated that the PI3K/Akt pathway is central in the development of intima hyperplasia after vascular injury *in vivo* [24][25]. Further we could show that vaspin has no effect on the migration and the proliferation of HUVEC. In contrast to the inhibitory effect of vaspin on the PI3K/Akt pathway in smooth muscle cell, vaspin has been shown to induce Akt activation in human aortic endothelial cells, protecting them thereby from free fatty acid-induced apoptosis [26]. This is of vital importance for the endothelializaion of the stent after implantation which determines the long-term outcome and the risk for acute stent-thromosis and myocardial infarction. In combination with our clinical data, this gives us strong evidence for the observed correlation between the development of in-stent restenosis and the pre-interventional plasma levels of vaspin in an individual patient. Further the observed strong inhibitory effect on HCASMC but not on HUVEC *in vitro*. The almost abolished serum induced cell migration of HCASMC could be an explanation for the fact that in patients with the highest plasma vaspin levels no in-stent restenosis occurred. Interestingly, it has been shown recently that vaspin has also direct effects on human macrophages and treatment with vaspin reduces an inflammatory phenotype of human macrophages by nuclear factor κB down-regulation and significantly suppresses oxidized low-density lipoprotein-induced foam cell formation. This was associated with significantly reduced intraplaque inflammation in an *in vivo* model that employed chronic infusion of vaspin into Apoe−/− mice (PMID 29891806).

The source of circulating vaspin in plasma is not fully understood. The highest tissue expression levels of vaspin were found in liver brain and skin compared to only modest expression in adipose tissue, spleen and low or non-detectable expression in bone marrow, muscle and kidney[27]. Interestingly, Sato et al was able to demonstrate that vaspin is expressed at high levels in atheromatous plaques in particular in foam cells in human coronary arteries. In contrast vaspin was not observed in human normal coronary arteries [28]. However, no data is available on the expression of vaspin in perivascular adipose tissue or vascular cells in vessels with intima hyperplasia.

Some limitations of the present study have to be acknowledged. As the primary endpoint of this study was angiographic restenosis, we included only patients that gave their informed consent for control angiography. However, we do not believe that selection bias plays a major role since these patients did not differ in respect to baseline characteristics and outcome from the total cohort of patients that had PCI with implantation of DES. Further, our study is necessarily of an observational nature. Accordingly, our results may be explained by unmeasured confounding factors. Therefore, we tried to control for baseline imbalances by multivariate modeling. The possibility of residual or undetected confounding is small but cannot be ruled out completely. In addition, in our study only first generation DES were analyzed, however biological mechanisms of ISR are similar between first and younger generations of DES. In addition, in our cohort ISR rates after implantation of Cypher or TAXUS stents were remarkable different. We therefore included stent type in the multivariate analysis and could demonstrate, that the association of vaspin plasma levels with ISR was independent of used stent.

In conclusion, determination of vaspin plasma levels before PCI might be helpful in the identification of patients with high risk for development of ISR after stent implantation. In addition, the selective effects of vaspin on smooth muscle cell migration could potentially be used to reduce ISR without inhibition of re-endothelialization of the stented segment.

## Abbrevations

PCI: Percutaneous coronary intervention
ISR: In-stent restenosis
SMC: Smooth muscle cells
HCASMC: Human coronary artery smooth muscle cell
DES: Drug eluting stents
Vaspin: Visceral adipose tissue-derived serpin
ELISA: Enzyme-linked immunosorbent assays
NIH: Neointimal hyperplasia
ECM: Extracellular matrix
